# What's in a name? The case for standardized nomenclature for mutualistic Mucoromycotina ‘fine root endophytes’

**DOI:** 10.1093/jxb/eraf210

**Published:** 2025-05-14

**Authors:** Nathan O A Howard, Victor H Rodriguez-Morelos, Lewis Allen, Pedzisai Chinoruma, Louis D Cohen, Grace A Hoysted, Anne D Jungblut, Isabella Lamb, Sara Moeskjaer, Flavia Pinzari, James Prout, Claire E Stanley, Jurriaan Ton, Alex Watts, Alex Williams, Tim Daniell, Alan Wanke, Sebastian Schornack, Silvia Pressel, Katie J Field

**Affiliations:** Plants, Photosynthesis and Soil, School of Biosciences, University of Sheffield, Sheffield S10 2TN, UK; Department of Life Sciences, Natural History Museum, Cromwell Road, London SW7 5BD, UK; Department of Life Sciences, Natural History Museum, Cromwell Road, London SW7 5BD, UK; Plants, Photosynthesis and Soil, School of Biosciences, University of Sheffield, Sheffield S10 2TN, UK; Department of Bioengineering, Imperial College London, London SW7 2AZ, UK; Department of Biology, Maynooth University, Co. Kildare, Maynooth, Ireland; Department of Life Sciences, Natural History Museum, Cromwell Road, London SW7 5BD, UK; Plants, Photosynthesis and Soil, School of Biosciences, University of Sheffield, Sheffield S10 2TN, UK; Plants, Photosynthesis and Soil, School of Biosciences, University of Sheffield, Sheffield S10 2TN, UK; Department of Life Sciences, Natural History Museum, Cromwell Road, London SW7 5BD, UK; Institute for Biological Systems, Council of National Research of Italy (CNR), Montelibretti, Rome, Italy; Plants, Photosynthesis and Soil, School of Biosciences, University of Sheffield, Sheffield S10 2TN, UK; Department of Bioengineering, Imperial College London, London SW7 2AZ, UK; Plants, Photosynthesis and Soil, School of Biosciences, University of Sheffield, Sheffield S10 2TN, UK; Plants, Photosynthesis and Soil, School of Biosciences, University of Sheffield, Sheffield S10 2TN, UK; Plants, Photosynthesis and Soil, School of Biosciences, University of Sheffield, Sheffield S10 2TN, UK; Plants, Photosynthesis and Soil, School of Biosciences, University of Sheffield, Sheffield S10 2TN, UK; Sainsbury Laboratory, University of Cambridge, Cambridge CB2 1LR, UK; Sainsbury Laboratory, University of Cambridge, Cambridge CB2 1LR, UK; Department of Life Sciences, Natural History Museum, Cromwell Road, London SW7 5BD, UK; Plants, Photosynthesis and Soil, School of Biosciences, University of Sheffield, Sheffield S10 2TN, UK; Lancaster University, UK

**Keywords:** Arbuscular mycorrhizal fungi, endomycorrhizal fungi, fine root endophytes, glomeromycotina, mucoromycotina, mycorrhizal symbiosis, nomenclature, symbiosis

## Abstract

Multiple names are used to describe the same group of root-associated fungi. Following the International Conference for Mycorrhizas 12, we propose unifying them as ‘ֹMFRE’ to improve clarity and consistency and distinguish them from similar groups.

**Arbuscular mycorrhizal (AM) fungi are a near-ubiquitous group of plant symbiotic fungi and have been the focus of much mycorrhizal research over the last 60 years (Koide and Mosse, 2004). However, a lesser known group of mycorrhizal fungi, the Mucoromycotina ‘fine root endophytes’ (MFRE), have garnered increasing research interest in recent years. This early branching lineage of fungi (Bidartondo *et al*., 2011; Field *et al*., 2015) was recently reclassified as belonging to the subphylum Mucoromycotina (Orchard *et al*., 2017a) rather than the Glomeromycotina which encompasses the AM fungi (or ‘coarse root endophytes’). Considering the rapidly growing interest in fine root endophytes, the use of a consistent nomenclature is becoming an important issue for the research community. Here, we summarize the background literature and recent discussion from the 12th International Conference on Mycorrhiza (ICOM12; 4–9 August 2024, Manchester, UK), proposing a standardized and cohesive terminology for this group of enigmatic, though widespread, endomycorrhizal fungi.**

Mucoromycotina ‘fine root endophytes’ have a somewhat obscure and limited recorded history because, until recently, they have been difficult to observe, identify, isolate, and culture. Endophytic fungi likely to have been Mucoromycotina ‘fine root endophytes’ were probably first identified in association with the evergreen tree species *Griselinia littoralis* (Kapuka, New Zealand broadleaf, or Pāpāuma, family—*Griseliniaceae*) by Greenall (1963) and named *Rhizophagus tenuis* owing to their morphological similarity to the AM fungal species *Rhizophagus populinus.* This similarity was based on the presence of arbuscule-like structures, though it was noted that the vesicles and hyphal diameters were both smaller than those of *R. populinus*. This endophyte also resisted attempts at axenic culture at the time of the study.

In the following decade, *R. tenuis* was mostly portrayed as being a type of AM fungus. References to fine root endophytes (‘FRE’) in the mycorrhizal literature simply indicated its likely presence in a sample (Baylis, 1967; Mosse and Hayman, 1971; Crush, 1973a). In 1973, Crush (1973b) reported the effects of *R. tenuis* colonization on the growth of three grass species under low phosphorus (P) conditions, where *R. tenuis* was shown to improve plant biomass, an effect that was reversed on fertile soils. This effect was confirmed by Johnson (1976), with fine root endophyte colonization of *G. littoralis* and *Leptospermum scoparium* (Mānuka or tea tree, family—*Myrtaceae*) resulting in a higher P concentration in plant dry matter produced on low-P soils compared with asymbiotic plants.

Since the early studies in the 1960s and 1970s, fine root endophytes have featured only occasionally in the mycorrhizal literature, with both field and laboratory studies focusing on their effects on plant hosts in terms of biomass. However, none of these studies measured carbon for nutrient exchange between symbiotic partners, merely recording the presence or absence of the fungus in samples based on morphology determined through optical light microscopy (Daft and Nicolson, 1974; Sainz *et al*., 1990; Postma *et al*., 2007). The occurrence of such nutrient exchange has now been confirmed to occur between Mucoromycotina ‘fine root endophytes’ and diverse vascular and non-vascular plant species (Field *et al*., 2016; Hoysted *et al*., 2023; Howard *et al*., 2024; Fig. 1).

**Fig. 1.**
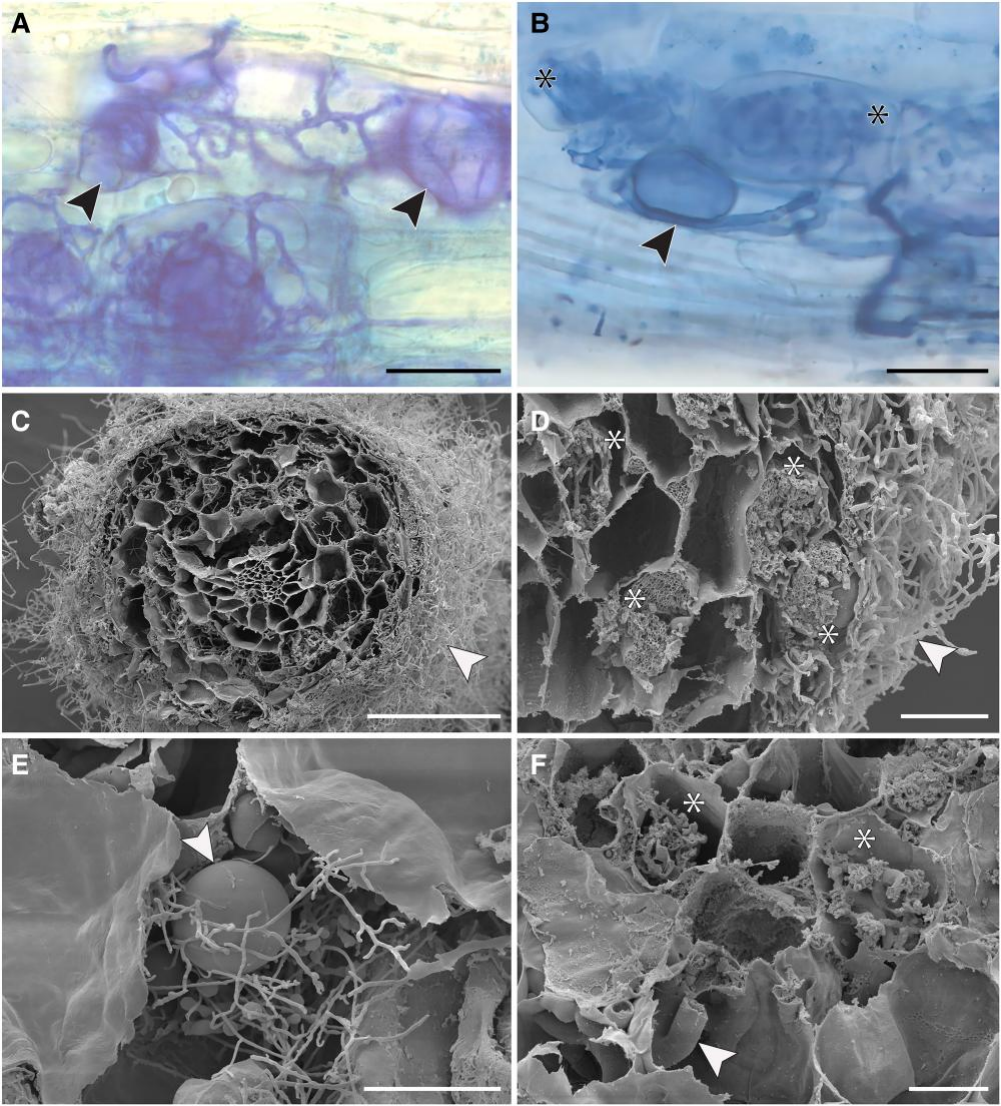
Fungal structures produced by MFRE isolates and the AM fungus *Rhizophagus irregularis*. Trypan blue-stained light micrographs (A and B) and scanning electron micrographs (C–F) showing fungal structures produced by MFRE isolates (A, C–E) versus those formed by the AM fungus *R. irregularis* MUCL 41833 (B and F) in roots of white clover (*Trifolium repens* L.) (B–F) and Ri T-DNA-transformed carrot (*Daucus carota* L.) roots established under *in vitro* culture conditions (A). (A) MFRE highly branching fine hyphae and vesicle-like structure (arrowed) in cells. (B) *R. irregularis* coarse hyphae (arrowed) next to a vesicle and arbuscules (*) in cells. (C) *T. repens* root heavily colonized by an MFRE fungus; note the ‘hyphal mantle’ enveloping the root (arrowed). (D) Cells packed with tightly wound hyphal coils (*). Abundant MFRE mycelium is tightly appressed to the root surface (arrowed) forming a ‘mantle-like’ structure. (E) MFRE fine branching hyphae and vesicle-like spherical structure (arrowed) inside a root cell. (F) *R. irregularis* coarse hypha (arrowed) and arbuscules (*) in root cells. Scale bars: (C) 100 µm; (A, B, D–F) 20 µm. (Image credits: original micrographs produced by Victor H. Rodriguez-Morelos and Silvia Pressel.)

*Rhizophagus tenuis* was reclassified as *Glomus tenue* by Hall (1977), based on morphological features distinct from other species of *Rhizophagus*, albeit also noting some physical differences between *G*. *tenue* and other members of the genus. This morphological distinction (Fig. 1), coupled with DNA analysis, allowed Orchard *et al*. (2017a) and Desirò *et al*. (2017) to conclude that *G. tenue* (or *tenuis*) belongs within the fungal subphylum Mucoromycotina, rather than Glomeromycotina which contains AM-forming species. Subsequently, a new genus, *Planticonsortium*, has been suggested for Mucoromycotina fine root endophytes (Walker *et al*., 2018) with the combination *P*. *tenue*. It remains unclear whether these fine root endophytes (formerly *G. tenue*) might represent more than one species, as suggested by Thippayarugs *et al*. (1999).

Most contemporary literature continues to use variations of the term ‘fine endophyte’ including ‘fine root endophyte’ (FRE), ‘Mucoromycotina fine root endophyte’ (MFRE), or ‘MucFRE’. These nomenclatures reduce ambiguity and maintain a clear distinction from Glomeromycotina AM-forming fungi. More recently, however, this group of fungi has been referred to as ‘Mucoromycotinian arbuscular mycorrhizal fungi’ (‘M-AM’ fungi, or ‘M-AMF’; Albornoz *et al*., 2022; Kowal *et al*., 2022). By conflating the fine root endophytes with AM fungi, the distinctiveness of the two groups of fungi is obscured in three critical ways. First, it departs from the conventions of all previous common names which included some reference to the morphology (‘fine endophytic’) of these fungi. If the term ‘M-AM’ fungi, or ‘M-AMF’, were adopted, all subsequent research on and reference to these fungi would be nominatively detached from the preceding work, hindering literature searches and concealing current knowledge from future research, further complicating an already complex history. Secondly, the use of the ‘AM fungi’ as part of this new term adds unnecessary taxonomic confusion as all AM-forming fungal species (excluding FRE/MFRE/MucFRE) belong to the Glomeromycotina subphylum (Orchard *et al*., 2017b). Referring to the fungi in question with a term already in use for species within a different taxonomic group adds unnecessary confusion and conflates the distinction between these separate fungal groups. Additionally, while arbuscule-like structures are sometimes observed in host plants colonized by Mucoromycotina ‘fine root endophyte’ fungi (Sinanaj *et al*., 2021; Hoysted *et al*., 2023), they are by no means ubiquitous or diagnostic of colonization by these fungi, and their function remains unconfirmed. It is clear that arbuscules are not required for bi-directional exchange of resources between plant hosts and Mucoromycotina fine root endophytes (e.g. Hoysted *et al*., 2023; Howard *et al*., 2024). Finally, the use of the term M-AM fungi (M-AMF) would necessitate the renaming of all other AM fungi species as Glomeromycotinian AM fungi (G-AMF) (Albornoz *et al*., 2022), the wide adoption of which is extremely unlikely to occur consistently in an already large and rapidly growing body of literature.

Therefore, based on phylogenetic evidence (Bidartondo *et al*., 2011; Rimington *et al.*, 2015, 2018; Desiró *et al.*, 2017; Orchard *et al*., 2017a), we propose that the more accurate term Mucoromycotina ‘fine root endophyte’ (‘MFRE’) is used to refer to the endosymbiotic fungi within the Mucoromycotina clade. This name is consistent with both the historical nomenclature and the currently known genetic identity of these fungi. ‘MFRE’ retains the historically used morphological description of ‘fine root endophyte’, showing a clear connection between early and more modern literature while further reducing possible conflation with AM fungi by the inclusion of ‘Mucoromycotina’. This term also avoids both the need to rename AM fungi in all future publications, and any reference to arbuscular structures that are not consistently present in these symbioses.

## Concluding remarks

Currently, several names are used to refer to the same group of mycorrhiza-forming soil fungi in the subphylum Mucoromycotina (Table 1). We believe this should be streamlined for consistency, clarity, and ease of understanding for the wider scientific community. We propose that the term Mucoromycotina ‘fine root endophytes’, ‘MFRE’, be adopted as representing a phylogenetically and morphologically accurate term that references the classic literature and minimizes confusion or conflation with the other common group of endosymbiotic mycorrhiza-forming fungi, AM fungi, which belong to the single fungal subphylum, Glomeromycotina.

**Table 1.** Summary of terminologies used to refer to endosymbiotic Mucoromycotina fungi

Name/abbreviation	Also known as	Key sources	Notes
*Rhizophagus tenuis*	Fine root endophytes (FRE) Fine endophytes (FE)	Greenall (1963)	Original description and naming of ‘fine endophyte’ in *Griselinia littoralis* roots. Classification conflates FRE with AM fungi.
*Glomus tenuis*		Gerdemann and Trappe (1974) Hall (1977)	*Rhizophagus tenuis* revised taxonomically and reclassified under *Glomus,* alongside AM fungi.
Mucoromycotina fungi		Bidartondo *et al*. (2011) Field *et al*. (2015, 2016) Rimington et al. (2018)	Identified as mutualistic endosymbionts in non-vascular plants. Evidence of diversity within Mucoromycotina fungi reflected in broad naming convention.
MFRE/MucFRE	Mucoromycotina fine root endophyte	Orchard *et al*. (2017a, b) Hoysted *et al.* (2018, 2021, 2023) Kowal *et al.* (2020) Sinanaj *et al*. (2021, 2024) Howard *et al.* (2022, 2024) Yang *et al*. (2024) Prout *et al*. (2024) Rosling *et al*. (2024)	Link between Mucoromycotina fungi and FRE confirmed. MFRE established as widely distributed nutritional mutualists in a variety of vascular plant species. Term effectively delineates MFRE from AM fungi and makes the link between Mucoromycotina fungi and fine root endophytes explicit.
*Plantiscortium tenue*		Walker *et al*. (2018)	Taxonomic reclassification from *Glomus tenuis* to *Plantiscortium tenue*. Term does not reflect diversity of MFRE which remains largely unknown.
M-AMF / M-AM	Mucoromycotinian arbuscular mycorrhizal fungi	Albornoz *et al*. (2022) Kowal *et al*. (2022) Mansfield *et al*. (2023) Seeliger *et al*. (2024) Liu *et al*. (2024)	Reversion to arbuscular mycorrhizal (AM) terminology to describe fine endophytes. Conflates the distinctions between fungal groups; adoption would require renaming of AM fungi.
